# Life-Course SES Disadvantage and Dual-Poverty Trajectories in Later Life: Evidence From China

**DOI:** 10.1093/geroni/igaf122.3013

**Published:** 2025-12-31

**Authors:** Ting Hu, Yu-Chih Chen

**Affiliations:** University of Illinois--Urbana-Champaign, Champaign, Illinois, United States; National Taiwan University, Taipei, Taipei, Taiwan (Republic of China)

## Abstract

Research on life-course socioeconomic status (SES) and health outcomes in later life has been widely examined, but the application on financial health remains underexplored. Furthermore, financial health measurement tends to be a single dimension focusing on income or assets. To comprehensively understand financial health, this study adopts an asset-based perspective to construct financial health using income and asset poverty as measurements. Guided by the life course models, we examined whether and how four life course models (latency, pathway, cumulative, and mobility) were associated with dual poverty trajectories measured by income and assets. Using 1,976 respondents aged 45 and older from five waves (2011–2018) of the China Health and Retirement Longitudinal Study, sequence analysis and multinomial regression were used to examine how SES risk in childhood and early adulthood was associated with dual-poverty trajectories in later-life. Four types of dual-poverty trajectories were identified through sequence analysis: persistent non-poor, persistent asset-only poverty, transitioning to non-poor from asset poverty, and those switched between income and asset poverty. Regression results showed that the relationship between early-life SES disadvantages and later-life poverty was partially mediated by the SES risk in early adulthood. Furthermore, individuals with persistent disadvantage or downward mobility showed higher risks of all types of poverty. Findings highlight the importance of addressing cumulative SES disadvantages in both early-life and adulthood stages. Policies that support education, improve financial literacy, provide financial assistance, and promote asset-building initiatives should be established to promote financial health and economic security in later life.

